# Second-harmonic generation tuning by stretching arrays of GaAs nanowires†

**DOI:** 10.1039/d2nr00641c

**Published:** 2022-06-13

**Authors:** Grégoire Saerens, Esther Bloch, Kristina Frizyuk, Olga Sergaeva, Viola V. Vogler-Neuling, Elizaveta Semenova, Elizaveta Lebedkina, Mihail Petrov, Rachel Grange, Maria Timofeeva

**Affiliations:** ETH Zurich, Optical Nanomaterial Group, Institute for Quantum Electronics, Department of Physics 8093 Zürich Switzerland gsaerens@phys.ethz.ch; ITMO University Kronverkskiy prospect 49 197101 St Petersburg Russia; DTU Fotonik, Technical University of Denmark 2800 Kongens Lyngby Denmark; NanoPhoton – Center for Nanophotonics, Technical University of Denmark 2800 Kongens Lyngby Denmark

## Abstract

We present a wearable device with III–V nanowires in a flexible polymer, which is used for active mechanical tuning of the second-harmonic generation intensity. An array of vertical GaAs nanowires was grown with metalorganic vapour-phase epitaxy, then embedded in polydimethylsiloxane and detached from the rigid substrate with mechanical peel off. Experimental results show a tunability of the second-harmonic generation intensity by a factor of two for 30% stretching which matches the simulations including the distribution of sizes. We studied the impact of different parameters on the band dispersion and tunability of the second-harmonic generation, such as the pitch, the length, and the diameter. We predict at least three orders of magnitude active mechanical tuning of the nonlinear signal intensity for nanowire arrays. The flexibility of the array together with the resonant wavelength engineering make such structures perspective platforms for future bendable or stretchable nanophotonic devices as light sources or sensors.

## Abstract 2

We present a wearable device with III–V nanowires in a flexible polymer, which is used for active mechanical tuning of the second-harmonic generation intensity.

## Graphical abstract

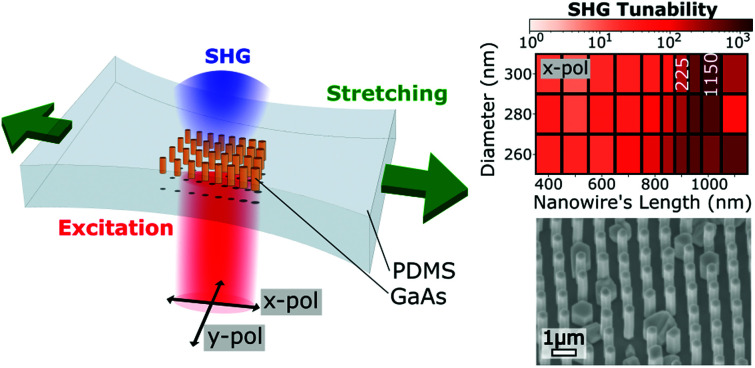

Compound semiconductor nanowires (NWs) offer a unique optical platform for various photonic applications including light emission in both linear and nonlinear regimes.^1–3^ The high refractive index of semiconductor NWs provides confinement and enhancement of electromagnetic fields without significant light absorption in the visible part of the spectrum and almost fully relieved of losses in the near infrared region.^4,5^ Direct band gap III–V semiconductor nanostructures such as GaAs are known as efficient sources of luminescent signal^3^ and possess a strong optical nonlinearity allowing for enhanced nonlinear optical processes including second-harmonic generation (SHG).^6–8^ The enhancement of the SHG signal has already been extensively studied in the past years. L. Carletti *et al.* designed AlGaAs nanoantennas to support Mie resonance at telecom band^9^ and V. F. Gili *et al.* measured in such structures SHG efficiencies around 10^−5^ GW cm^−2^.^10^ In the recent work of D. de Ceglia *et al.* a similar SHG conversion efficiency can be expected for a single GaAs NW by resonantly coupling the pump and the second-harmonic (SH) fields.^11^ S. Liu *et al.* demonstrated how overlapping magnetic dipoles in a NW array leads to an enhancement of the SHG conversion efficiency by four orders of magnitude in comparison to an unpatterned GaAs thin film.^12^ While passive systems based on semiconductor nanoantennas and metasurfaces have already shown very effective light conversion and emission, the dynamical control and tunability of their optical properties is highly desirable for real-time modulation of optical signals. One of prospective directions are mechanically tunable systems for which their optical properties can be reconfigured by applying a mechanical force.^13,14^ GaAs NWs are good candidates for various optomechanical systems as being obtained with a bottom-up or a top-down fabrication approach, they still can be released using wet etching,^15^ peeling off,^16^ transfer printing^17^ or mechanical exfoliation^18^ methods. By integrating them with various flexible media, several applications have been demonstrated as bendable or stretchable light sources,^15,18^ sensors,^19,20^ lenses,^21^ electronic components,^22,23^ optical filters^24,25^ or photodetectors.^25,26^

While flexible optical structures have been utilized for tuning the linear optical properties of dielectric systems,^15,18–26^ the mechanical control over the nonlinear signal generation has not been demonstrated yet in such structures. The development of novel systems for flat nonlinear optics will bring more functionalities with an eye-safe pumping scheme in the near infrared and flexible frequency conversion at different wavelengths. 2D metastructures such as metasurfaces, metalenses, and metagratings have already demonstrated their efficiency in optical frequency conversion^27^ and diffraction^28^ control, and even in the generation of entangled photon pairs.^29^

Results of our work will open prospective for fine nonlinear modulation of high-*Q* resonances sensitive to particular geometries such as quasi bound-states-in-the-continuum modes.^30,31^ Alternatively, the nonlinear optical devices are usually operating in high-power laser regimes, which inevitably leads to heating of the systems. In this way, tunable mechanical stretching structures, demonstrated in this study, can compensate for the thermal drift of the resonances driven by thermos-refractive effects. In this work, we show how to overcome the fixed geometry constraint for efficient light conversion with mechanical active tuning of the SHG intensity in NW arrays embedded into a flexible polymer film. The NWs were grown on a silicon substrate by metalorganic vapour-phase epitaxy and detached from the substrate with a sharp blade. We achieved up to 30% uniaxial tensile stretching and a mechanical tunability of the SHG by a factor of almost two. The SHG conversion efficiency at 0% stretching was around 10^−9^ W^−1^. These experimental results were further supported with simulations of SHG conversion efficiency tunability of an uniaxially stretched array of NWs with different lengths and diameters and for an incoming polarization parallel or perpendicular to the stretching direction. We provide guidelines with range of design parameters that can be targeted to obtain a maximised tunability. The calculations predict maximum values of three orders of magnitude with theoretical SHG conversion efficiency up to 10^−3^ W^−1^. We believe these hybrid features of a NW array within a stretchable matrix are convenient for further applications as light sources or light sensors in biology or as key components for flexible and wearable display technologies.^32–34^

First, we provide the modelling used for the linear and nonlinear optical response of a 2D NW array. Calculations were performed with a finite element model for an ideal periodic structure. The rectangular unit cell is composed of polydimethylsiloxane (PDMS) and of a single GaAs NW with specific length *L* and diameter *D* in the centre (Fig. 1a). At rest, the side lengths of the square unit cell are equal (Fig. 1b). Under uniaxial stretching one side length of the rectangular unit cell is varied (*x*-direction), while the other is fixed (*y*-direction), see also Fig. 1c. The pitch *p* is defined as the distance between neighbouring NWs in *x*-direction (see Methods for more details).

**Fig. 1:**
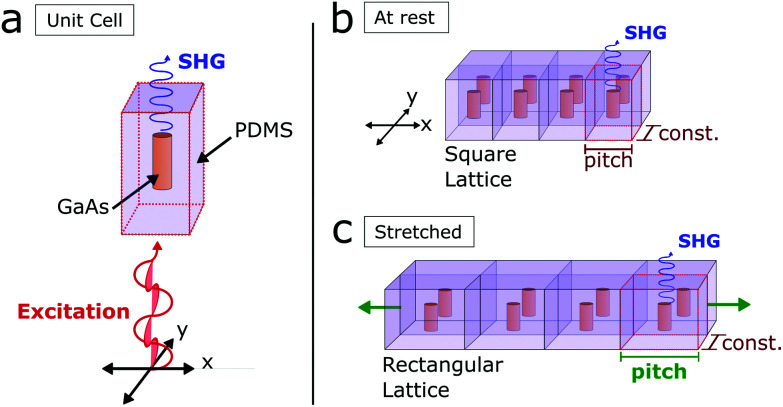
(a) Schematic of the periodic structure that is implemented in the finite element model. It consists of a unit cell with PDMS and a NW of specific length and diameter in the centre. (b) NW array at rest configuration and (c) under uniaxial stretching, for which the size of the unit cell varies only in one direction. A plane wave with linear polarization (x- or y-polarization) excites the GaAs NWs, generating SH.

The SHG conversion efficiency *σ* is calculated with 1/*W* units as the generated SH peak power divided by the excitation peak power squared *σ* = *P*(2*ω*)/*P*^2^(*ω*), similarly as in the work of M. Celebrano *et al.*^35^ The optical properties are governed by the lattice modes of the NWs array formed by the Mie resonances of individual cylinders. These modes impact greatly the SHG conversion efficiency but depend on the NWs length and diameter, on the pitch and on the excitation polarization. The band structure of the modes and the corresponding electric field distributions for a square lattice with a pitch of 600 nm (see Fig. 2a) show four branches with preferable x- and y-polarization directions and a degeneracy at the centre of the Brillouin zone (Γ-point). The modes 1 and 2 play the most important role as their *Q*-factor value obtained in numerical modelling is around *Q* ∼ 300. Modes 3 and 4 are low *Q*-factor modes with *Q* ∼ 5 and so do not contribute into the overall field enhancement (see Fig. S1 in the ESI†). This can also be seen in the field enhancement spectra (see Fig. 2b), where a single peak is observed exactly at the wavelength corresponding to the Γ-point of modes 1 and 2.

**Fig. 2:**
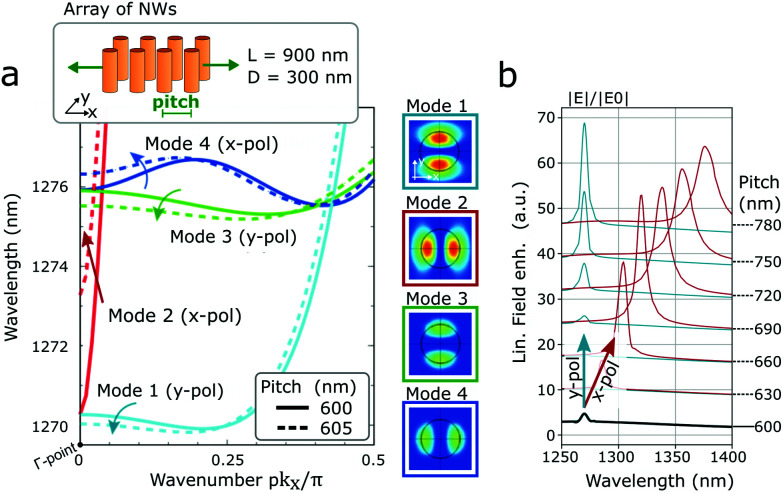
(a) Photonic band structure in an array of NWs with length *L* = 900 nm and diameter *D* = 300 nm. Dispersion of modes contributing to the transmission and reflection spectra are shown for a square grating with pitch *p* = 600 nm (solid line) and for a rectangular grating with pitch *p* = 605 nm (dotted line). The light “line” is almost vertical in the current wavelength's range, having coordinate p*k*_x_/π ∼ 0.95, and is not show in the figure. The inset shows the electric field distribution demonstrating x- and y-polarization of the modes. (b) Linear field enhancement, expressed as the ratio of electric field intensity inside the structure to the incoming field power. The resonance red-shifts only for x-polarization similarly as predicted from the band structure.

By stretching along the *x*-direction, which results in a pitch increase from 600 nm to 605 nm, the degeneracy at the Γ-point is split and the frequency of the modes are shifted. However, mode 2 (x-pol) has a significantly steeper dependence on the *k*-vector than mode 1 (y-pol) resulting in a different sensitivity to perturbations along the *x*-direction and, thus, a different spectral shift: only 0.3 nm for mode 1, while for mode 2 the shift is on the order of 3 nm (see Fig. 2a). This is in perfect agreement with the simulations of the field enhancement spectrum, expressed as the ratio of the electric field intensity inside the structure to the incoming field power (see Fig. 2b). We observe a strong spectral shift for an excitation with x-polarization (corresponding to mode 2) and a weak spectral shift for an excitation with y-polarization (corresponding to mode 1). Further analysis of modes is presented for a NW array in section S1 (see Fig. S2–S4†) and for a single NW in section S2 (see Fig. S5 and S6) in the ESI.† The NWs have a smaller or similar height compared to the wavelength (in the range of 500 to 900 nm), and possess a 1st and 2nd order Mie mode along the long axis (see Fig. S6 and S7 in the ESI†). Additional simulations for the impact of the NWs length on the resonance in the linear regime can be seen in Fig. S8 in the ESI.† With the further increase of the NW length the Mie modes will be modified into waveguide-type Fabry–Perot modes which can also provide additional enhancement of SHG *via* the phase matching condition well studied in the literature.^36,37^

The SHG conversion efficiency spectra are shown in Fig. 3a and b for an array of NWs with length *L* = 900 nm, diameter *D* = 300 nm and pitch *p* from 600 nm to 780 nm. These values are similar to the sample of NWs array that will be presented later in the Experimental section. At rest configuration, the spectrum shows a dominant lattice resonance around *λ* = 1270 nm (blue curve in Fig. 3a and b). This does not depend on the incoming polarization excitation being in the *x* or *y* direction as the periodic structure is symmetric under a 90° rotation along the light propagation axis. The variation of the pitch in one direction with x-polarization excitation leads first to an increase and then a decrease in the amplitude of the lattice resonance (Fig. 3a). Additionally, the position shifts for the x-polarization due to the overlap of the individual Mie resonances of the single NW elements (see Fig. S2 in the ESI†). An SHG conversion efficiency tunability can be calculated as *G* = *σ*_s_/*σ*_0_, where *σ*_s_ and *σ*_0_ are the SHG conversion efficiencies measured at the same wavelength but at different pitches, between *p* = 630 and *p* = 780 nm for the first (stretched) and at pitch *p* = 600 nm for the latter (at rest configuration). Around the lattice resonance a maximum value of *G* ≈ 225 for the excitation with x-polarization is found, while for the excitation with y-polarization this value is *G* ≈ 17. The tunability factor *G* was calculated (given with a logarithmic colour scale) for arrays with NWs of different lengths *L* and diameters *D*, as shown in Fig. 3c and d. For NWs with length around *L* = 1000 nm and diameter around *D* = 300 nm, the value of *G* is three orders of magnitude for the x-polarization (see Fig. 3c). Similarly, for NWs with lengths *L* = 900 nm and diameter *D* = 260 nm, the value of *G* is two orders of magnitude for the y-polarization (see Fig. 3d).

**Fig. 3:**
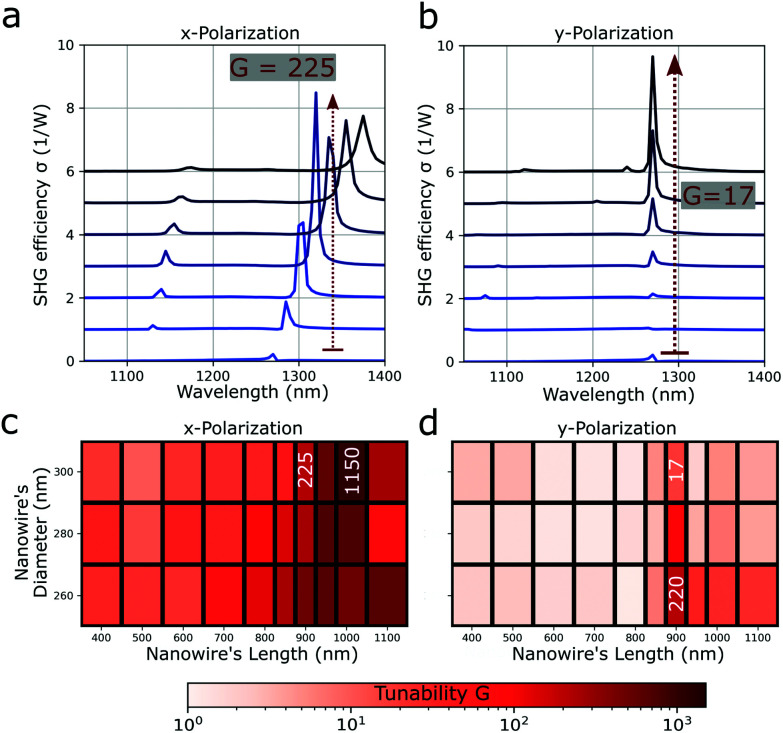
Simulated SHG conversion efficiency spectrum for an array of NWs with length *L* = 900 nm and diameter *D* = 300 nm is given for different pitch values from *p* = 600 nm (at rest configuration) to *p* = 780 nm (30% stretched). These values correspond also to the samples array of the experiments. Nonlinear efficiency calculated for an excitation (a) with x-polarization, (b) with y-polarization. The maximum nonlinear signal tunability for x-polarization is *G* = 225, and for the y-polarization is *G* = 17. (c and d) Calculated *G* values for different NWs lengths and diameters, plotted in a logarithmic scale. For an excitation with x-polarization, *G* is at least three orders of magnitude for slightly longer NWs, while with y-polarization, *G* is at least two orders of magnitude for slightly thinner NWs.

The higher values for an excitation with x-polarization can be explained by the red-shift of the lattice resonance as the SHG intensity at rest is almost zero at the resonance position of the stretched case. We believe even higher values can be reached by optimizing the pitch and varying with finer steps the NWs length and diameter. NWs shorter than 800 nm do not show high *G* values because the dominant lattice resonance is broader than for longer NWs.

We present experimental results that support the above simulations. The different fabrication steps of a stretchable periodic structure are schematized in Fig. 4a. A dielectric mask was used on silicon to grow arrays of NWs with metalorganic vapour-phase epitaxy (MOVPE). Scanning electron microscope images of the NWs were taken before a layer of flexible PDMS material was deposited (see Fig. 4b). Embedded NWs were finally mechanically extracted using a razor blade. More details about the fabrication procedure are given in Methods (see also section S3 in the ESI†). The flexible structure of NWs allows mechanical tensile stretching up to 30%, which is limited by the size of the sample compared to the setup. The SHG conversion efficiency was measured in transmission with the excitation wavelength in the region from *λ* = 1050 nm to *λ* = 1350 nm. The setup is shown in Fig. 4c and described in Methods.

**Fig. 4:**
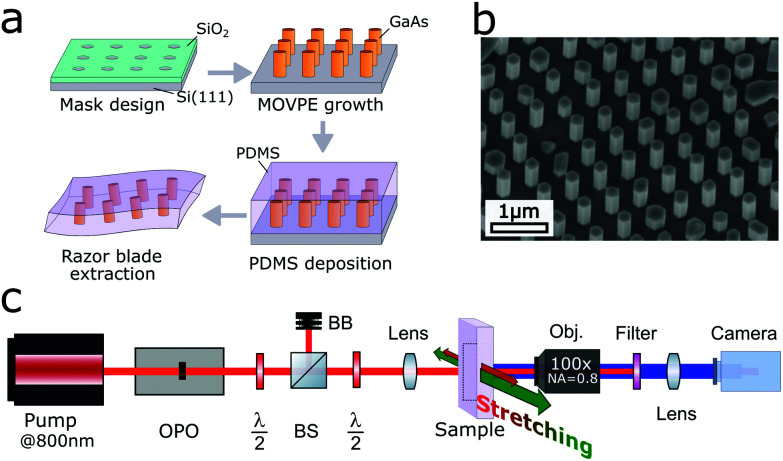
(a) Schematized fabrication steps of the stretchable sample. A SiO_2_ mask was fabricated, NWs were grown selectively on silicon with MOVPE, a layer of flexible PDMS material was deposited and embedded NWs were finally mechanically extracted using a razor blade. (b) Scanning electron microscope image of a GaAs NWs array selectively grown on Si. We measured a pitch of *p* = 600 nm. (c) Schematic transmission setup to measure the SHG conversion efficiency. OPO: optical parametric oscillator, *λ*/2: half-wave plate, BS: beam splitter, BB: beam block. The first *λ*/2 and the BS are used to select the power, while the second *λ*/2 is used to rotate the polarization.

Two regions containing short NWs (around *L* = 500 nm) and long ones (around *L* = 900 nm) were selected for linear and nonlinear optical characterization (see Fig. S8 in the ESI†). The experimental results for the transmission show good agreement with the simulation results, as shown in section S4 of the ESI.† The SH signal was measured at 10 different spots in each of the two regions (see Fig. 5a and b) and for an excitation with x-polarization (solid line) and y-polarization (dotted line). The characterization of the SHG signal is shown in section S5 of the ESI.† The spectra at rest (blue) are similar for the two polarizations, which is expected as the array is symmetric with respect to a 90° rotation along the light propagation axis. The resonance is broader than in the above simulations due to the not completely optimized physical bottom-up growing process that led to a non-negligible distribution of NWs lengths and diameters, which we could evaluate from SEM images. By measuring this local distribution and extrapolating numerically the SHG efficiency from arrays of NWs with different lengths and diameters, we calculated a single spectrum of the SHG conversion efficiency (see Methods and Fig. S8 and 9 in the ESI†). Fig. 5c shows this calculated spectrum for a region with shorter NWs, respectively Fig. 5d shows for a region with longer NWs. The simulations indicate a bigger tunability of the conversion efficiency for longer NWs than for shorter ones, as well as a red-shift of the resonance to *λ* = 1260 nm for the x-polarization. These two specific aspects are also visible in the experimental results shown in Fig. 5b.

**Fig. 5:**
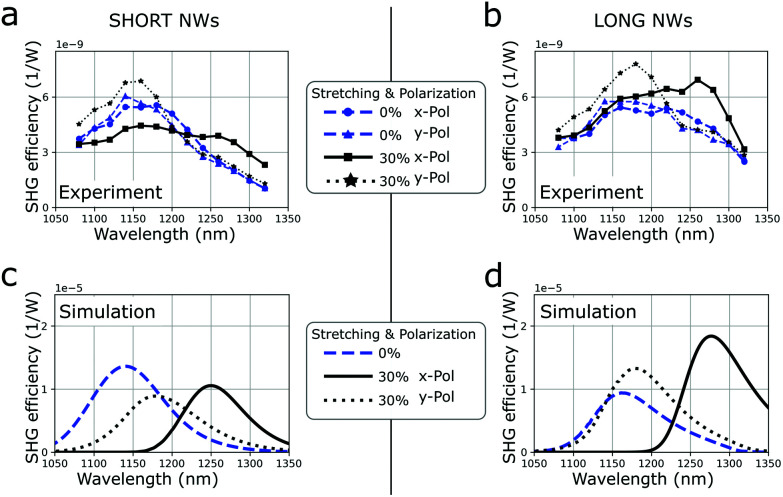
Experimental results of measured SHG conversion efficiency spectrum for the two regions of (a) short (around *L* = 500 nm) and (b) long (around *L* = 900 nm) NWs. In blue, measured when at rest and in black when stretched by 30%. The SHG conversion efficiency spectrum was measured at 10 different spots for the x-polarization (solid line) and y-polarization (dotted line). (c and d) Simulation results calculated taking into account the specific distribution of NWs size. For longer NWs, an increase in the SHG efficiency as well as peak position shift for an excitation with x-polarization was visible in the experimental and simulations results.

The measured tunability of the SHG conversion efficiency is not as strong as calculated with our simulation model. This can be explained by the sample imperfections as the NW arrays possess a significant distribution of sizes and also 10% of not-fully grown NWs (see Fig. 4b and Fig. S8 in the ESI†). In the simulations, the infinitely periodic structure was excited uniformly and the SHG was collected with all angles in the forward direction. However, in the experiment, the beam had a radius around 5 μm, exciting around 100–200 NWs, and the objective only collected in forward direction with a numerical aperture of 0.8. Another important aspect to be mentioned is that the interaction of NWs with different sizes was not considered in the simulations, while in the sample, NWs with slightly different lengths were located next to each other. We measure the SHG conversion efficiency like this *σ* = *P*(2*ω*)/*P*^2^(*ω*) = 10^−9^ W^−1^, or equivalently as a unitless efficiency *P*(2*ω*)/*P*(*ω*) = 10^−6^ for an average excitation power of 5 mW cm^−2^. The SH power *P*(2*ω*) measured after the sample and the excitation power at the sample *P*(*ω*) take into account the transmission losses from the optics. This experimental SHG conversion efficiency value is similar to single nanostructures or periodic arrangements.^12,38–40,45^

In conclusion, we demonstrate how arrays of GaAs NWs can be efficiently used to mechanically tune the SHG intensity in the near infrared. We fabricated a periodic structure based on patterned GaAs NWs embedded in PDMS. Our numerical calculations show that a tunability of the SHG conversion efficiency of at least 3 orders of magnitude is possible for an excitation polarization along the uniaxial stretching direction. Experimental results show an SHG tunability of almost 2 for 30% mechanical stretching and our developed numerical model indicate that the NWs size distribution is reducing the nonlinear optical performances. We calculate the SHG conversion efficiencies and tunabilities for an array with NWs of specific lengths and radiuses and believe these values can be used as a guideline for stretching capabilities of GaAs NWs arrays. Experimental improvements to obtain stronger resonances are possible by optimizing the epitaxial growth, or using a top-down approach. The SHG tunability can further be increased by applying a bigger mechanical stretching, especially for an excitation with y-polarization, or by stacking different layers of flexible NWs array on top of each other.

## Methods

### Nanowire growth

A 60 nm thick SiO_2_ mask was fabricated. GaAs nanowires were grown on pre-patterned Si (111)-oriented wafer in a low-pressure (80 mbar) MOVPE Turbodisc® reactor with hydrogen as a carrier gas. The growth temperature was 750° C. Trimethylgallium with flow rate of 7.8 × 10^−6^ mol min^−1^ and arsine with flow rate of 2.23 × 10^−3^ mol min^−1^ were used as group III and group V precursors, respectively. The pitch between the NWs was around 600 nm, their radius was around 130 nm, while their length varied from 500 nm (NWs in the centre) to 1300 nm (NWs in the edge) with a local distribution in the length of ±100 nm.

### Sample fabrication

Polydimethylsiloxane (PDMS) was fabricated from mixing a base and an agent with mass ratio 10 : 1 (SYLGARD® 184, silicone elastomer kit, Dowsil). We added Toluene (ACS reagent >99.7% (GC), Fluka) to change the density of the polymer (20% of total mass) and to achieve better stretching properties. After pouring the PDMS, we waited 10 min to allow the PDMS to penetrate between the NWs. The PDMS was spin coated (1000 rpm, 1000 rpm, 3 min) to form a thin layer embedding the grown NWs. It was heated to 80 °C for 1 hour and cooled for 3 hours. After that the thin layer with NWs inside was mechanically detached from the Si substrate by using a razor blade. An additional layer of PDMS was dropped on top and spin coated (100 rpm, 1000 rpm, 10 min) to form a large flexible sample. It was heated to 80 °C for 1 hour, cooled for another 3 hours and a square (25 cm × 10 cm) was cut out with a blade.

### Optical measurements

The SHG intensity spectrum was measured with a fully-automated homemade nonlinear microscope system as illustrated in Fig. 4c. A pulsed light with tunable wavelength was generated by a Ti:Sapphire laser system (Chameleon Ultra II, Coherent) combined with an optical parametric oscillator (Compact OPO, Coherent). The pulses wavelength was tuned from 1050 nm to 1350 nm with a duration typically around 200 fs, a repetition rate of 80 MHz and an average power at the sample of 5 mW. The beam was focused to a beam radius of 5 μm on the sample with a lens (A240TM with NA = 0.5, Thorlabs) and collected with a 100× objective (LMPlanFL N with NA = 0.8, Olympus). The sample was firmly attached on both sides and stretching was achieved by translating the two sample holders in opposite directions (precision of translation is 0.1 mm for a 15 mm rest length). The signal was focused with a convex lens (la1461 with *f* = 250 mm, Thorlabs) onto a sCMOS camera (Zyla 4.2, Andor) and the SH signal was separated using two high-pass filters.

### Simulation model

Calculation were performed with a finite element method (COMSOL Multiphysics). An infinite array was defined through its unit cell which consisted of a rectangle out of PDMS material (refractive index around *n*(*ω*) ≈ 1.4), with perfectly matched layers on top and bottom, and a GaAs NW in the middle (refractive index around *n*(*ω*) ≈ 3.4). The bottom side of the unit cell was set as an entry port for a plane wave excitation while the top side was a collection port for the SHG intensity. The electric field for the linear scattering regime was calculated for an incoming linearly polarized plane wave with an intensity of 30 GW cm^−2^, which corresponded to the peak power at the sample in the experimental setup. The induced nonlinear polarization was given by the *χ*^(2)^ tensor for zinc-blende GaAs, for which the biggest component *χ*_36_ = 370 pm V^−1^. We can neglect the stretching of the NWs as the Young's modulus of PDMS^41^ is 4 orders of magnitude lower than the one of GaAs.^42^ A detailed mathematical description of the implemented code to obtain the induced nonlinear polarization and then the intensity of the SH is given with a different geometry in our previous work.^43,44^ Since the NWs were grown along the (111) direction, the tensor was also rotated following tensor rotation calculations.^44^

### Accounting for NWs size distribution

We developed another approach for which we fitted the main resonance from the simulation spectrums with a Gaussian peak and extrapolated the amplitude, the position and the width for arrays of NWs with different lengths and diameters. It was then possible to come up with a single and smoother approximation spectrum by adding the extrapolated Gaussian peaks with their extrapolated parameters as well as with the corresponding weight given from the (continuous) size distribution (see also Fig. S9 in the ESI†).

## Author contributions

The manuscript was written through contributions of all authors. M. T., R. G. and M. P. designed the experiment. M.T. and G. S. built the setup and conducted the nonlinear experiments. M. P., O. S, K. F., E. B. and G. S. performed the different numerical simulations. E. L. and E. S. fabricated the arrays of GaAs NWs. G. S. prepared the flexible sample with the help of M. T. and V. V.-N. M. T., R. G., M. P. and G. S. analysed the data. V. V.-N. and G. S. performed the transmission measurements. M. T., R. G., M. P. and G. S. wrote the manuscript. All authors have given approval to the final version of the manuscript.

## Conflicts of interest

There are no conflicts to declare.

## Supplementary Material

NR-014-D2NR00641C-s001
