# Supplementary material for: Second-harmonic generation tuning by stretching arrays of GaAs nanowires
Source: Nanoscale. 2022 Jun 13;14(24):8858–64. doi: 10.1039/d2nr00641c (PMC9219810; doi:10.1039/d2nr00641c)
Supplement: NR-014-D2NR00641C-s001 [file NR-014-D2NR00641C-s001.pdf]

Electronic Supplementary Material (ESI) for Nanoscale.
This journal is © The Royal Society of Chemistry 2022

Supporting Information

Second-Harmonic Generation Tuning by Stretching Arrays of GaAs Nanowires

Grégoire Saerens[1,], Esther Bloch[1], Kristina. Frizyuk[2], Olga Sergaeva[2], Viola V. Vogler-Neuling[1], Elizaveta Semenova[3,4], Elizaveta Lebedkina[3], Mihail Petrov[2], Rachel Grange[1], Maria Timofeeva[1]*

[1] ETH Zurich, Optical Nanomaterial Group, Institute for Quantum Electronics, Department of Physics, 8093 Zürich, Switzerland
[2] ITMO University, Kronverkskiy prospect 49, 197101 St. Petersburg, Russia
[3] DTU Fotonik, Technical University of Denmark, 2800 Kongens Lyngby, Denmark
[4] NanoPhoton–Center for Nanophotonics, Technical University of Denmark, 2800 Kongens Lyngby, Denmark

*Corresponding author: gsaerens@phys.ethz.ch

S1. Details on field distributions and resonances of Nanowire arrays

We first show in Figure S1 the Q-factor resonance corresponding to Figure 2 in the main text. Mode 1 & 2 possess much stronger Q-factors of around 300 than Mode 3&4. Mode 3 and 4 are low Q-factor modes with Q ~ 5 and so do not contribute into the overall field enhancement.

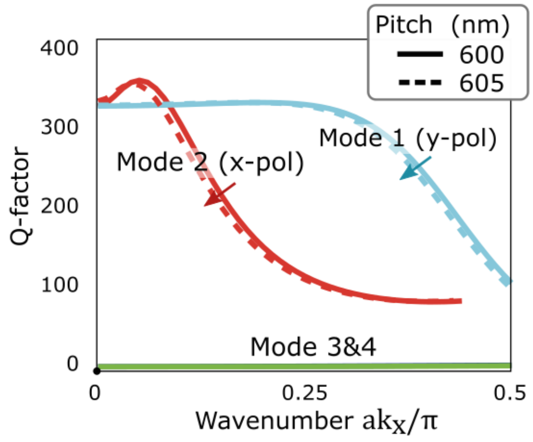

Figure S1. Q-factor corresponding to the band diagram shown in Figure 2 in the main text. Mode 1 & 2 possess much stronger Q-factors of around 300 than Mode 3 & 4 with Q ~ 5.

We also study the optical resonance supported in an array of NWs with length L = 900 nm, diameter D = 300 nm for which the second-harmonic generation (SHG) tunability under stretching is strongest. The linear transmission spectrum and the SHG conversion efficiency spectrum for this NWs array are given in Figure S2a-b. The resonance at λ_{res} = 1270 nm wavelength indicated by the arrow is visible in the transmission and SHG spectrum. In Figure S2c-d we plotted the electric and magnetic field intensity distributions $|E(2\omega)|^2$ and $|H(2\omega)|^2$ (normalized to the incoming electric field) at a resonant wavelength of λ_{res} = 1270 nm and a non-resonant wavelength of λ_{nr} = 1240 nm. The second-harmonic (SH) electric field is the strongest at the edges of the NW while the SH magnetic field is the strongest inside the NW. Inside the NW at resonant wavelength, we can observe five nodes in the SH electric field distribution and six nodes in the SH magnetic field distribution, which may indicate a multipole or Fabry-Perot resonance.

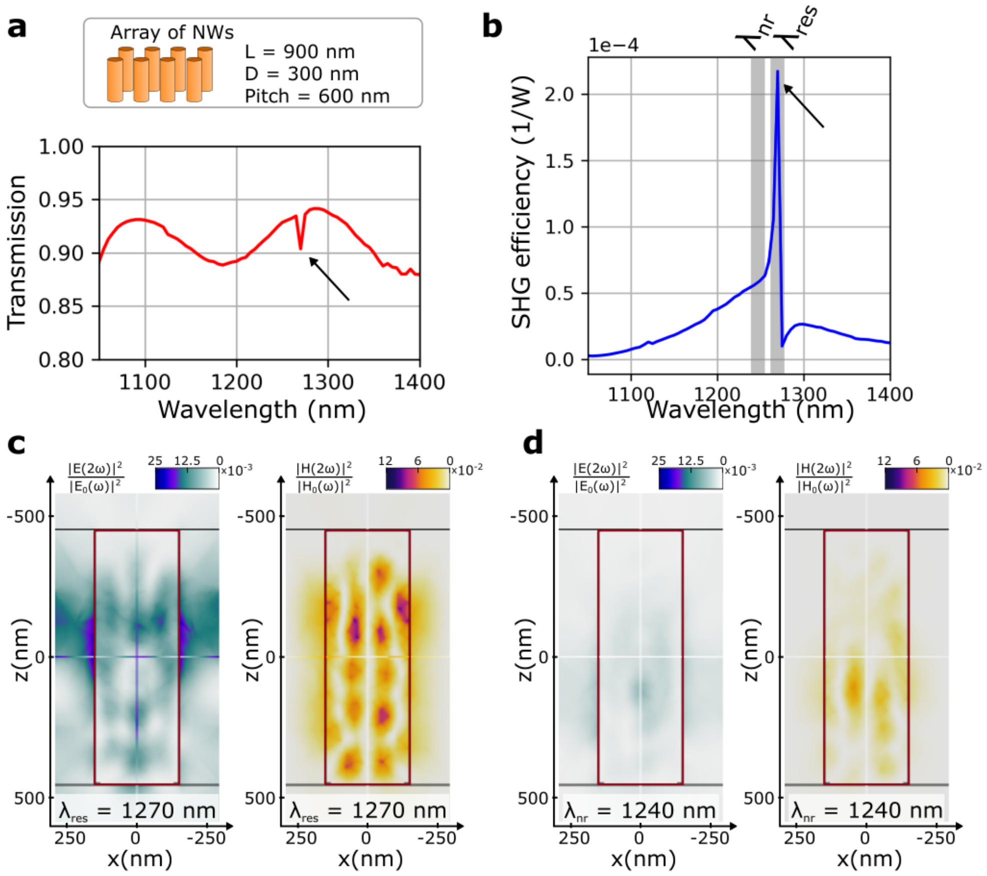

Figure S2. (a) NW Array with transmission spectrum and (b) SHG conversion efficiency. A resonance for high SHG generation is found at wavelength 1270 nm. (c,d) Relative intensity enhancement of the generated SH electric and magnetic fields at resonant wavelength λ_{res} = 1270 nm and out of resonance at λ_{nr} = 1240 nm. Five nodes in the SH electric field distribution and six nodes in the SH magnetic field distribution are visible inside the NW.

In Figure S3, we compare the linear electric field distributions (normalized to the incoming electric field) at rest configuration, when the pitch p = 600 nm, (see Figure S3a,b) with the fields at high resonance when the pitch p = 690 nm for x-polarization (see Figure S3c,d) and p = 780 nm for y-polarization (see Figure S3e,f). The field distributions are shown at the xy-plane with the NW in the centre. We observe in the stretched case the same linear electric field distributions (Figure S3b,d,f) as shown in Figure 2a with mode 1 (y-polarization) and mode 2 (x-polarization).

The electric field corresponding to mode 2 at resonance (see Figure S3d) is also distributed in y-direction to the neighbour NW, indicating a strong coupling between NWs.

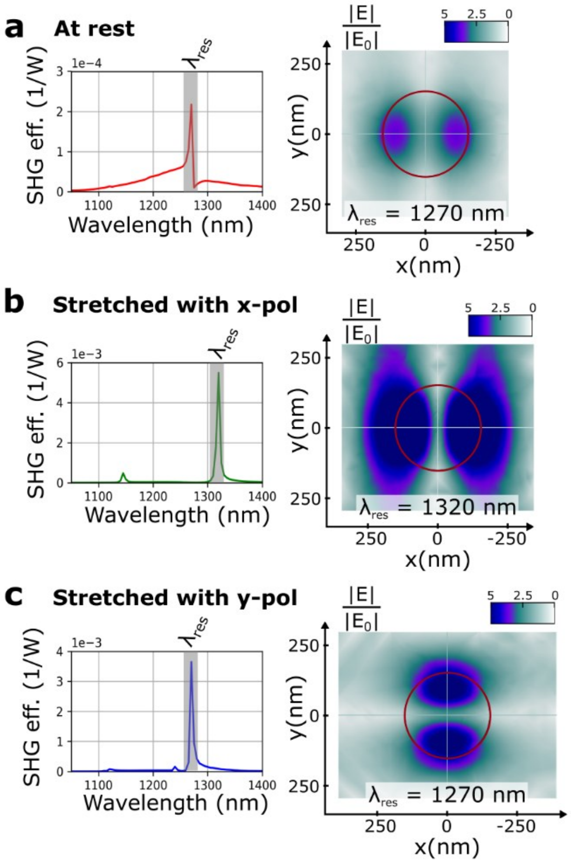

Figure S3. (a) SHG conversion efficiency spectrum for an array of NWs with length L = 900 nm, diameter D = 300 nm and pitch p = 600 nm. (b) At the resonant position λ = 1270nm, we plot the electric field distribution normalized to the incoming field. It is shown in xy-plane with the NW contour delimited by the red circle. (c-d) Similar plots for maximum resonance with x-polarization, which is for a pitch p = 690 nm and (e-f) for y-polarization which is for a pitch p = 780 nm. Field distribution as similar as in Figure 2a in the main text.

We computed the SHG conversion efficiency of an array of NWs with length L = 900 nm, diameter D = 300 nm and pitch p = 600 nm for which we changed only one parameter in order to observe the impact on the resonance. The length L is varied from 600 nm to 1100 nm, the diameter

D from 260 nm to 300 nm and the whole structure is rescaled from 85% to 115% (the length, diameter and pitch are simultaneously changed by this factor) (see Figure S4). An increase in length or diameter will red-shift the resonant wavelength position and tune the intensity of the resonance (see Figure S4a,b), while rescaling the system will shift the resonance but preserve the maximum resonance intensity (see Figure S4c). It is possible to choose these three parameters to tune the resonant intensity and wavelength position.

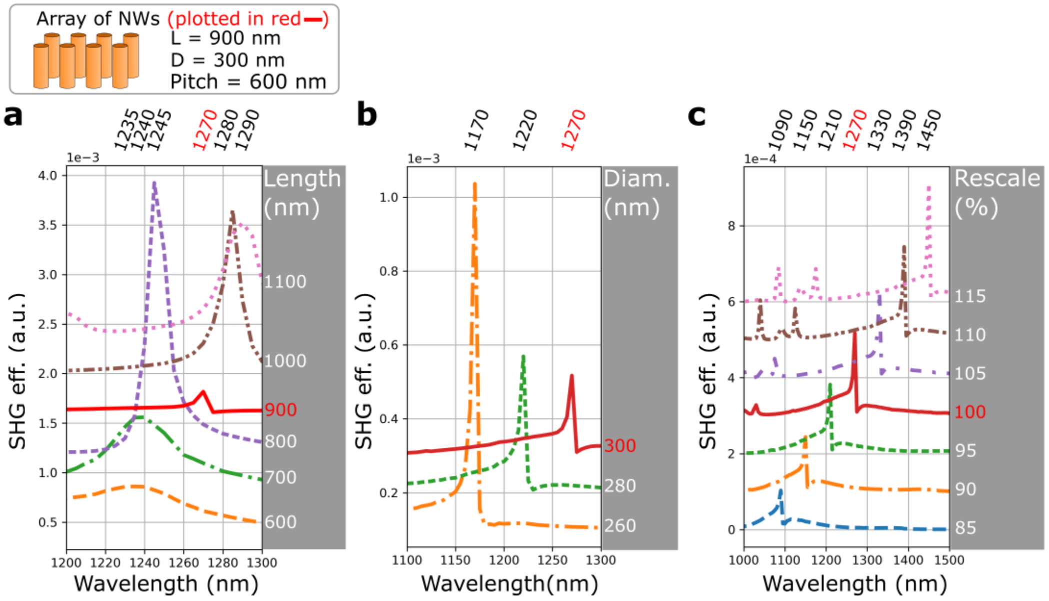

Figure S4. Resonance shift and intensity variation in an array of NWs for which we tuned (a) the length from 600 nm to 1100 nm, (b) the diameter from 260 nm to 300 nm and (c) rescaled the system from 85% to 115% (the length, diameter and pitch simultaneously by the same factor). An increase in length or diameter changes the resonance intensity and red-shifts its position, while rescaling the system only shifts the resonant position without affecting the intensity of the resonance.

S2. Details on field distributions and resonances of single NWs

The previous simulations, as well as all simulations for an infinite array in this manuscript, are performed for a rectangular unit cell geometry with specific boundary conditions (Floquet periodicity)(see Figure S5a). In this section we are interested in a single building block element of the array. The numerical simulation to compute the spectrum and multipole decomposition of a single NW is performed with a spherical geometry (see Figure S5b). Yet, the steps to compute the linear scattering and the SHG spectrum (with multipole decomposition) are always the same in all our simulation codes, see Figure S5c.

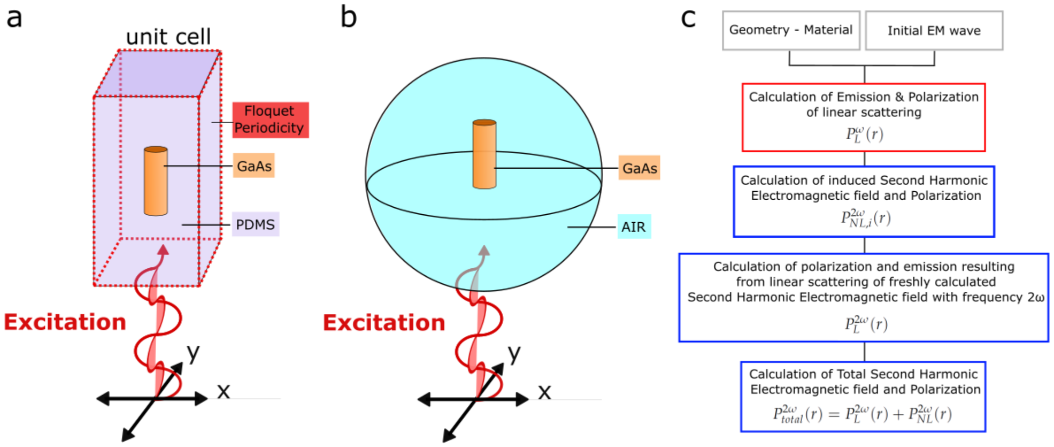

Figure S5. Geometry to compute the linear and nonlinear signal from (a) an array of NWs and (b) a single NW. The infinite boundary conditions for the array are defined for a Floquet periodicity. (c) Steps to compute the linear scattering and the SHG spectrum.

We compute with the finite element method simulations the scattering intensities and the multipole decomposition for a single nanowire (NW) with two different geometries of length L = 500 nm and diameter D = 260 nm (Figure S6) and of length L=900 nm and diameter 300 nm (Figure S7).

We observe that for the smaller NW around a wavelength of 1100 nm the electric dipole (ED) and the magnetic dipole (MD) are dominant, while for the second NW, the ED and the MD dominate around 1400 nm. The spatial field distribution intensities of the electric $|E|^2$ and magnetic $|H|^2$ fields are shown in Figures S6b,c and S7b,c for both NWs at the wavelength corresponding to the dipole dominance. We can confirm this dominance by looking at the field nodes in the structure as we observe in Figures S6,7 two nodes in the electric field distribution (at the top and bottom of the NW) and three nodes in the magnetic field distribution (top, bottom and around the middle of the NW).

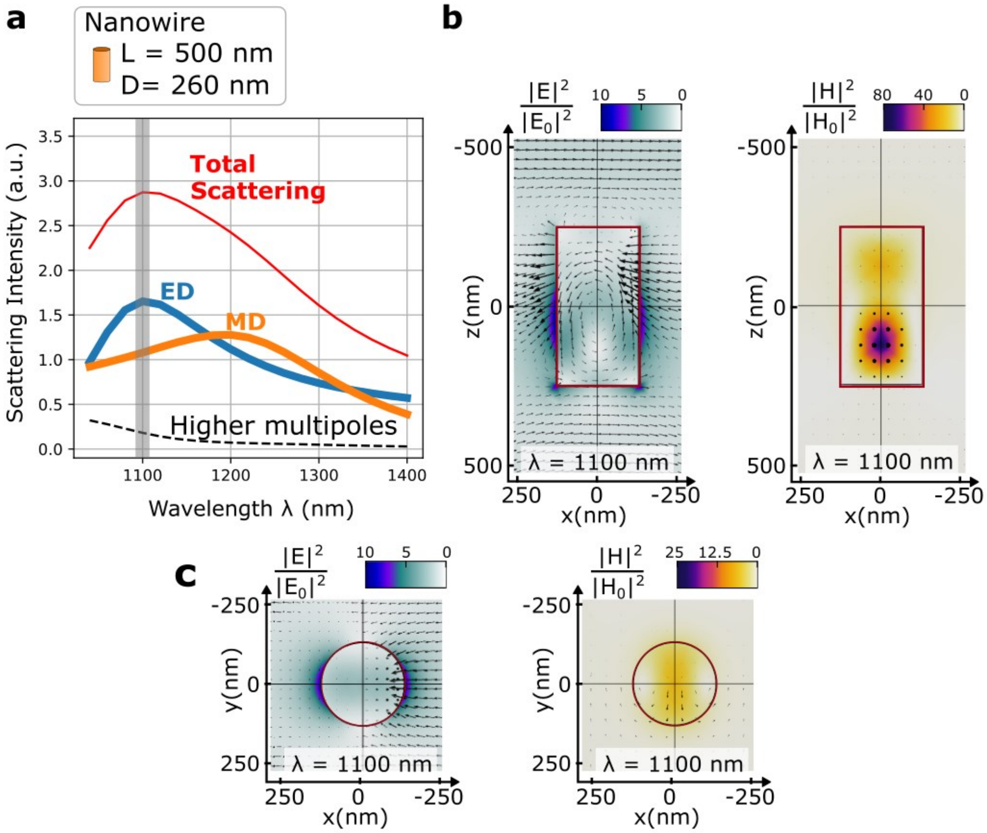

Figure S6. (a) Linear scattering spectrum of a NW with length 500 nm and diameter 260 nm. The NW is excited with a plane wave propagating in z-direction and polarized linearly in x-direction. The ED and MD dipoles are dominant around the wavelength 1100 nm. (b) Spatial field

distribution intensities of the electric $|E|^2$ and magnetic $|H|^2$ fields shown in the xz-plane. The color scale gives the relative field enhancement and the black arrows are projections of the vectorial field. (c) Similar top view of spatial field distribution intensities. The shape of the NW is given with the red rectangle/circle. At 1100 nm, two nodes are present in the electric field distribution and three nodes in the magnetic field distribution, which indicates the dipole dominances.

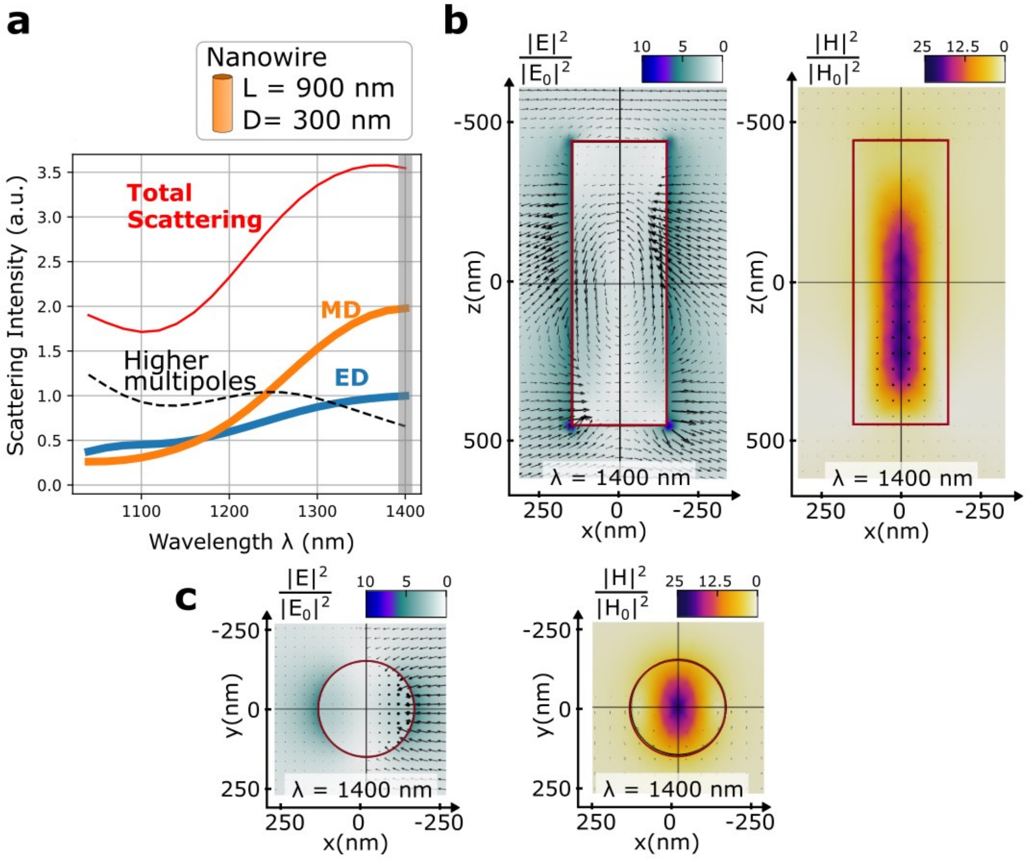

Figure S7. (a) Linear scattering spectrum from a single nanoantenna NW with length 900 nm and diameter 300 nm. The NW is excited with a plane wave propagating in z-direction and polarized linearly in x-direction. The ED and MD are dominant around the wavelength 1400 nm. (b) Spatial field distribution intensities of the electric $|E|^2$ and magnetic $|H|^2$ fields shown in the xz-plane. The color scale gives the relative field enhancement and the black arrows are projection of

the vectorial field. (c) Similar top view of spatial field distribution intensities. The shape of the NW is given with the red rectangle/circle. At 1400 nm, two nodes are present in the electric field distribution and three nodes in the magnetic field distribution, which indicates the dipole dominances.

To study the impact on the resonance of single NWs length, we calculate the linear scattering intensity for a nanowire with diameter D=260 nm and different lengths, see Figure S8a. The resonance shifts to longer wavelengths as the length of the NW increases. The multipole decomposition, shown in Figure S7b as a percentage of electric and magnetic dipole, reveals that these types of multipoles are dominating for NWs with length above 700 nm.

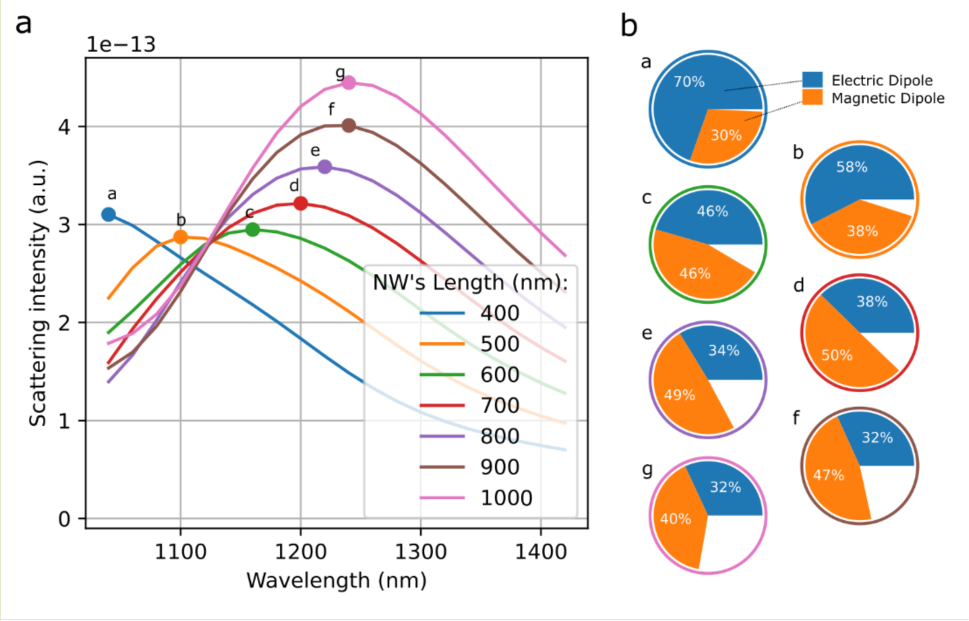

Figure S8. (a) Linear scattering spectrum from a single nanoantenna NW with different lengths. The resonance shifts to higher wavelengths for longer NWs. (b) Percentage at resonance of electric and magnetic dipole obtained from the multipole decomposition. Higher orders are not negligible anymore for a nanowire with length above 800 nm.

S3 Detail on sample with NWs distribution of size

The fabrication procedure is described in the manuscript (see also Figure 4a). We show here the sample after metalorganic vapour-phase epitaxy (MOVPE) (see Figure S9a), after the Polydimethylsiloxane (PDMS) deposition (see Figure S9b) and after mechanical extraction with a Razor blade (see Figure S9c). We also capture two images to show the extracted NWs. The first Image, shown in Figure S9d, is obtained with a 5x objective (under light illumination), the second image, shown in Figure S9e, is obtained with a 100x objective (the contrast was expressed with a yellow/purple colormap). We can clearly observe with the 100x objective the periodicity of the structure given by the NWs. More details about the fabrication procedure are given in Methods.

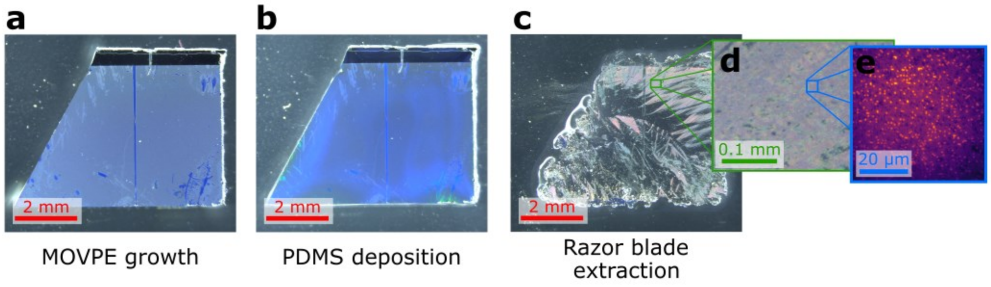

Figure S9. (a-c) Sample fabrication steps as given in Figure 4a from main text. Zoom in a region with extracted NWs obtained (d) with a 5x objective (under light illumination) and (e) with a 100x objective. The periodicity of the structure given by the NWs is clearly visible with the 100x objective.

The NWs composing the flexible photonic structure have varying sizes that we can describe with a distribution. For that we analysed several SEM images taken after MOVPE growth of the NWs and measured the lengths, diameters of the NWs and the pitch as the distance between NW centres. We observe that all NWs have the same diameter distribution but that NWs close to the edge are longer than in the middle of the sample. We can use this to characterize two regions where NWs

have different lengths but similar diameters. The distributions are shown in Figure S10a-b for the two areas, with respectively a SEM image in the inset. The counts are described with a Weibull function as it fits best the asymmetric Gaussian-like distributions. The mean length for the shorter NWs is 560 ± 100 nm, while for the longer NWs it is 930 ± 125 nm. We can also observe that around 10% of NWs are not fully grown. The results for the radius are shown in Figure S10c and do not show a significant difference for different spots, the mean radius is 133 ± 18 nm.

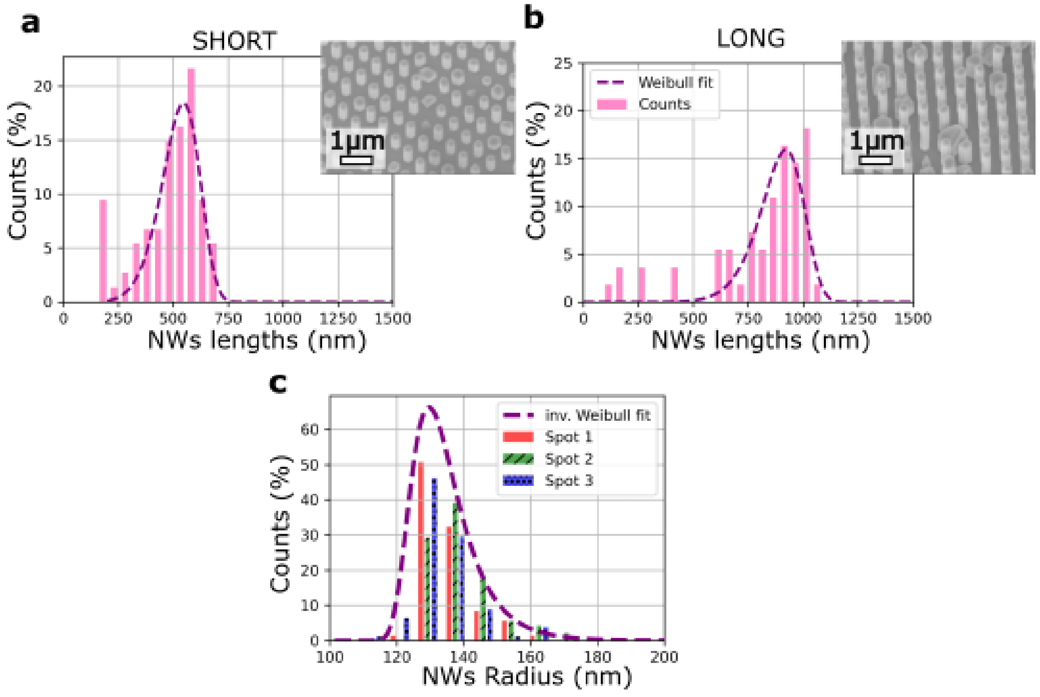

Figure S10. Distribution of length for the region with a) short NWs (around 500 nm length) and b) long NWs (around 900 nm length). Insets show the corresponding scanning electron microscopy images used to calculate the distribution of sizes. A Weibull function was taken to fit the distribution. c) Distribution of NWs radius shown for three different spots. The distribution is fitted with an inverted Weibull function. The distribution of diameter is constant over the whole sample.

The model used to compute into a single spectrum the SHG efficiency of an array with NWs of different sizes is explained in the Method section and illustrated here. Due to the not completely optimized bottom up growth method for the NWs, their heights and diameters varied locally as shown in Figure S10. A direct comparison between experimental results and simulations was not adequate as the latter was done for an ideal sample with NWs of unique height and diameter. As a first approximation, we calculated the discrete probability of having a NW with a certain size from corresponding SEM images, and added together the simulated spectrums of the ideal cases with their respective discrete weights. However, for the nonlinear SHG conversion efficiency spectrums, the IR resonance was too narrow and it would have needed a lot of simulations to avoid an unrealistic saw-like spectrum. Therefore we developed another method to come up with a single spectrum. Each SHG efficiency spectrum possesses a main peak that is fitted with a Gaussian function. We extracted for arrays with NWs of different lengths and diameters the amplitude (Figure S11a), the position (Figure S11b) and the width (Figure S11c) of this peak. The comparison between the reconstructed spectrum (in color, continuous line) and the original spectrum (in grey, dotted line) is shown for different arrays in Figure S11d. We came up with the single spectrum as Figure 5c,d in the main text by summing up the extrapolated spectrum with the corresponding probability distribution of the array with NWs the given length and diameter (see Figure S11e). This can be expressed like this: $\sum_{\text{Length, Diam}} \mathit{Distribution} \cdot \mathit{Amplitude} \cdot e^{-\left(\frac{\lambda - \mathit{Position}}{\mathit{Width}}\right)^2}$.

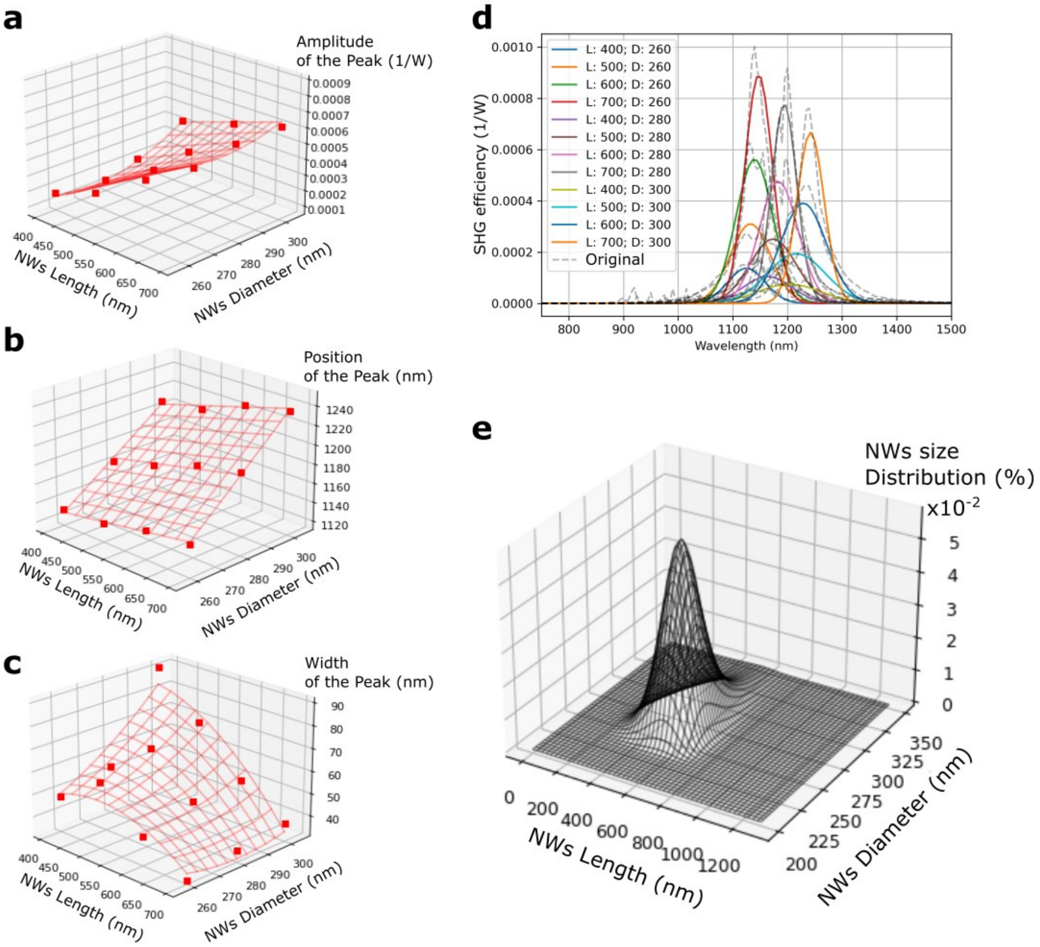

Figure S11. The SHG efficiency spectrum were fitted with a Gaussian function and (a) the amplitude, (b) position and (c) width of the main peak was recorded and extrapolated for every length and diameter of the NWs. (d) Comparison between the reconstructed spectrum (in color, continuous line) and the original spectrum (in grey, dotted line). (e) The probability of finding a NW with specific length and diameter for a specific region that was imaged with SEM.

S4. Transmission measurement

We measured the linear transmission spectrum for the two different regions with short (around 500 nm length) and long NWs (around 900 nm length). The setup used here is the same one as used for this other linear measurement.[3] To simulate the transmission curve for the sample

possessing a non-negligible distribution of sizes (NWs length and diameter), we summed up with the corresponding weight the transmission curves for an array of NWs with specific length and diameters. The experimental results and the simulation calculations are shown in Figure S12. We observe good matching in both cases as the shorter NWs have a large deep in transmission around 1100 nm while the longer NWs have a deep around 900 nm. The lower transmission observed experimentally can be explained by absorption and scattering in the PDMS or by the interaction of NWs with different sizes, which is not taken into account by the simulations model.

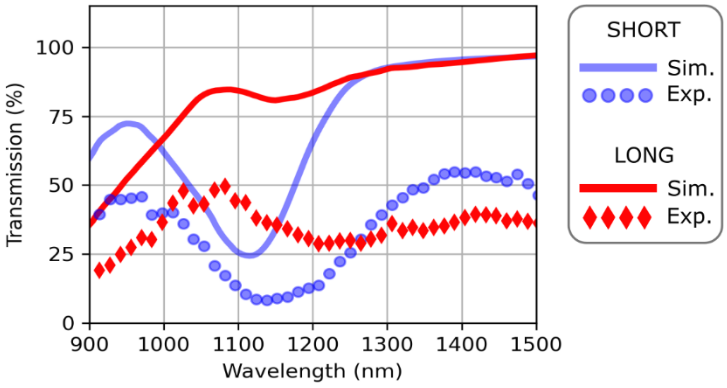

Figure S12. Experimental and simulation results of the transmission for two different regions on the sample with shorter (circle-blue) and longer NWs (diamonds-red). The experimental and simulation results agree well as the shorter NWs possess a transmission deep around 1100 nm while the longer NWs possess a transmission deep around 900nm.

S5. Characterization of SHG signal

We provide an extensive characterization of the signal measured experimentally. We can observe a speckle pattern with the camera for every spot and both excitation polarization, see Figure S13a for x- and y-polarization. The Count rate recorded by the camera is above 10^3 1/s per pixel. The power dependence of the signal is also quadratic as expected for SHG signals (see Figure S13b).

Figure S13c shows the spectrum of the excitation laser (in red), this excitation spectrum artificially frequency doubled (dotted) and the collected SHG signal (in blue). We clearly do not observe fluorescence from the sample.

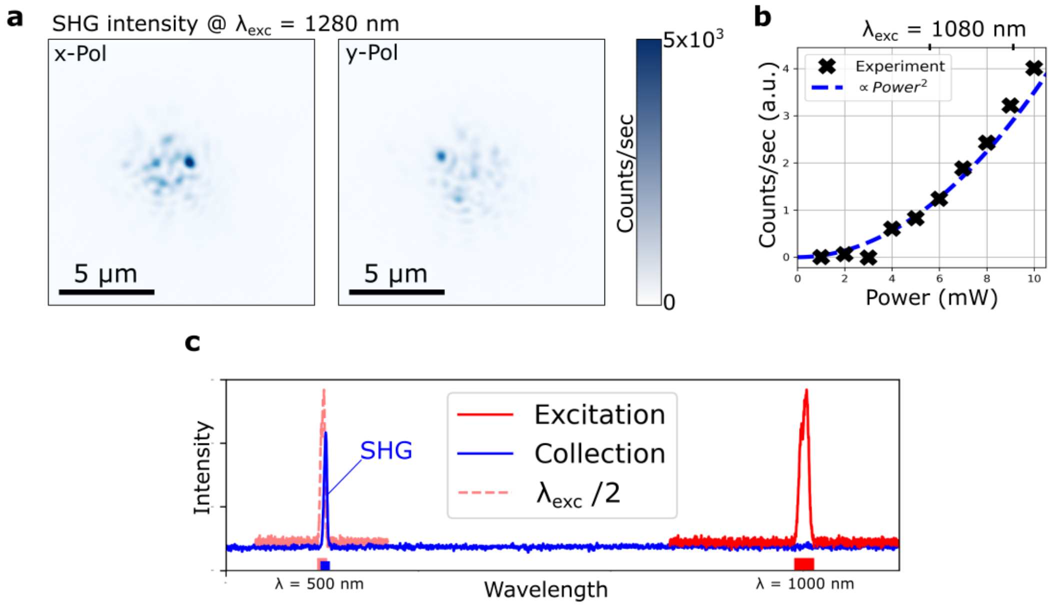

Figure S13. (a) Filtered image of the signal for the two polarizations: x-polarization and y-polarization. The camera counts (without any corrections) are on the order of 10^3 1/s. (b) Power dependence of the signal compared to a quadratic curve and (c) intensity spectrum of the excitation laser compared to the filtered signal. We clearly identify the signal as SHG.

References

(1) Timofeeva, M.; Lang, L.; Timpu, F.; Renaut, C.; Bouravleuv, A.; Shtrom, I.; Cirlin, G.; Grange, R. Anapoles in Free-Standing III-V Nanodisks Enhancing Second-Harmonic Generation. *Nano Lett.* **2018**, *18* (6), 3695–3702. https://doi.org/10.1021/acs.nanolett.8b00830.

(2) Saerens, G.; Tang, I.; Petrov, M. I.; Frizyuk, K.; Renaut, C.; Timpu, F.; Reig Escalé, M.; Shtrom, I.; Bouravleuv, A.; Cirlin, G.; et al. Engineering of the Second-Harmonic Emission Directionality with III–V Semiconductor Rod Nanoantennas. *Laser Photonics Rev.* **2020**, *14* (9), 1–10. https://doi.org/10.1002/lpor.202000028.

(3) Weigand, H.; Vogler-Neuling, V. V.; Escalé, M. R.; Pohl, D.; Richter, F. U.; Karvounis, A.; Timpu, F.; Grange, R. Enhanced Electro-Optic Modulation in Resonant Metasurfaces of Lithium Niobate. *ACS Photonics* **2021**, 1–6. https://doi.org/10.1021/acsphotonics.1c00935.
